# Quantitatively Profiling the Evolution of Hydrogen Storage and Defect Healing Processes in Palladium at the Nanoscale

**DOI:** 10.1021/acsnano.4c16841

**Published:** 2025-03-04

**Authors:** Yu-Cheng Chiu, Bo-Yi Chen, Chin-Chia Hsu, Chia-Wei Tsai, Shih-Ming Wang, I-Ling Chang, Chih-Wei Chang

**Affiliations:** † Center for Condensed Matter Sciences, National Taiwan University, Taipei 10617, Taiwan; ‡ Department of Mechanical Engineering, National Cheng Kung University, Tainan 70101, Taiwan; § Center of Atomic Initiative for New Materials (AI-MAT), National Taiwan University, Taipei, 10617 Taiwan

**Keywords:** hydrogen storage, palladium, defects, light elements, electron microscopy

## Abstract

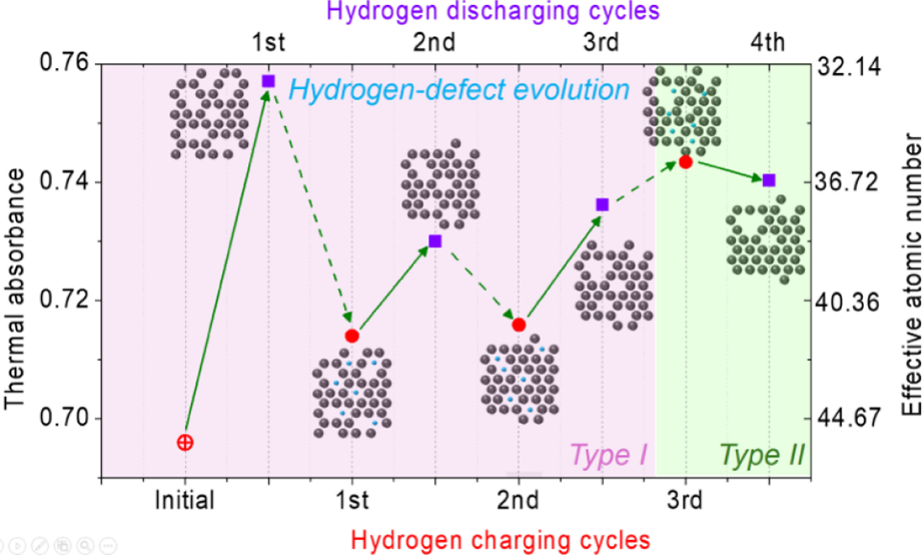

Light elements or compounds with an average atomic number (*Z*) of less than 10 are difficult to detect due to their weak interactions with electrons and photons. Here, we introduce a direct thermal absorbance measurement platform for scanning electron microscopy. The technique, named ZEM, is particularly sensitive to low *Z* materials, including hydrogen (*Z* = 1) and vacancy (*Z* = 0). We use Pd as an example to explore ZEM’s potential in characterizing hydrogen storage materials. ZEM reveals that hydrogen storage is highly inhomogeneous, concentrating on grain boundaries and defects. ZEM also unveils a large defect density created by hydrogenation, uncovering abundant voids beneath the surface. ZEM’s nondestructive detection method allows us to investigate multiple hydrogen charging–discharging cycles, revealing two distinct hydrogen uptake phenomena accompanied by unusual defect healing processes. We further establish the causality between hydrogenation and defect formation, quantifying distinct correlations between hydrogen-induced defect generation and defect-mediated hydrogen trapping. The rich phenomena discovered by the ZEM underscore its potential in material characterizations, particularly for light elements or compounds.

## Introduction

One of the fundamental challenges in developing a hydrogen economy is reducing the technical barriers to characterizing hydrogen-related phenomena.^1,2^ These include the physics and chemistry of hydrogen embrittlement,^3,4^ hydrogen trapping at interfaces or defects,^5,6^ and hydrogen spillover occurring at the nanoscale.^7−10^ However, very few tools are available to meet the requirement.^11,12^ Sievert or gravimetric techniques employ pressure or weight measurements to determine hydrogen absorption/desorption of bulk materials and generally do not exhibit spatial resolution. Thermal desorption spectroscopy (TDS) can detect hydrogen desorption at sub-ppm levels but cannot distinguish the signals’ spatial variations.^11,12^ Neutron scattering can probe the structure of hydrides or hydrogen diffusion dynamics using diffraction techniques, but it cannot characterize samples smaller than millimeters.^11^ Secondary ion mass spectroscopy (SIMS) can localize the hydrogen in a material with ∼100 μm lateral resolution and ∼0.1 μm depth resolution.^13^ However, because SIMS is a destructive method, it cannot be used to study hydrogen evolution for multiple charging–discharging cycles. Atom probe tomography (APT) requires dedicated sample preparation procedures to improve the spatial resolution of hydrogen detection to the nanoscale, but it also relies on a destructive method.^5,6,14^ On the other hand, because one can easily focus an electron beam beyond the diffraction limit of light, material characterizations at the nanoscale are usually carried out by various electron microscopy techniques. However, standard tools like electron probe microanalysis equipped with wavelength dispersive X-ray spectroscopy cannot detect elements with *Z* < 3.^15,16^ Fundamentally, the weak interaction between hydrogen atoms and the probe makes nanoscale characterization difficult.

Compared to techniques for detecting the hydrogen content, even fewer tools are capable of quantifying the vacancy concentration. Archimedes’ method relies on microbalance and only applies to bulk samples.^17^ The differential thermal expansion method relies on precise measurements of 3(Δ*L*/*L* – Δ*a*/*a*) where Δ*L*/*L* is the temperature-dependent thermal expansion, and Δ*a*/*a* is lattice constant variation.^18^ It has been a technique exclusive to bulk materials until recently.^19^ On the other hand, vacancy detection based on positron annihilation spectroscopy (PAS) relies on the longer lifetime of positrons when interacting with vacancies.^20^ However, the estimation of vacancy concentration relies on the parameters of trapping rate models and accessing a focused positron beam has remained challenging. Thus, the spatial resolution of PAS could reach ∼50 μm so far.^21^

**Figure 1:**
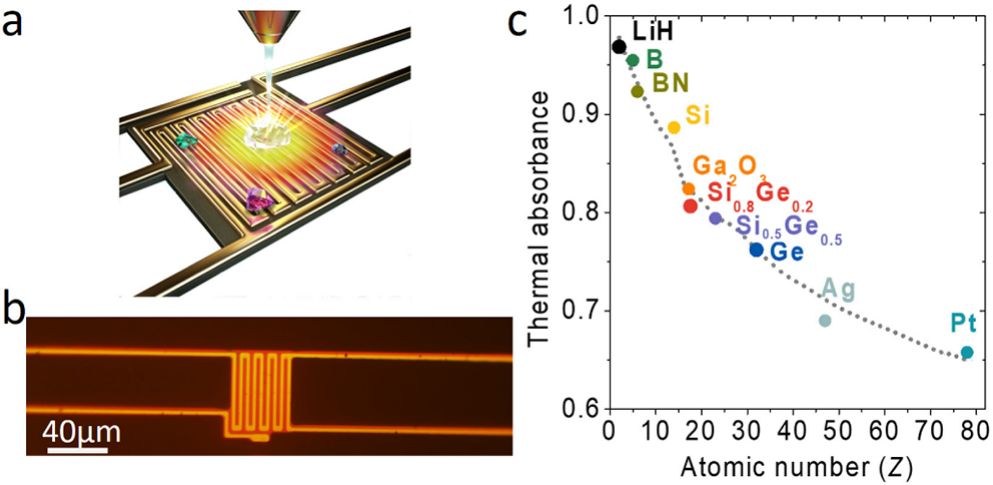
Direct thermal absorbance measurement platform and the operation of ZEM. (a) A schematic figure of a direct thermal absorbance measurement platform with samples inspected under an electron beam. (b) An optical image of the platform consisting of a patterned Pt thermometer supported by suspended SiN_*x*_ beams. (c) The experimental data of *A*_th_ (solid color symbols, obtained from our earlier work^23^). The results of the CASINO simulation for *A*_th_ (red dash-dotted curve) under a *V*_acc_ = 5 kV electron beam are also shown for comparison.

Here, we address both challenges using a single new technique. As shown in Figure, 1(a and b), we have recently introduced a direct thermal absorbance measurement platform within a scanning electron microscope (SEM) and discovered that the majority of the absorbed energy is converted to heat, i.e., *A*_th_/*A* = 98%,^22,23^ where *A* is the absorbance and *A*_th_ is the thermal absorbance. Because other forms of absorbance, such as X-ray, cathodoluminescence, or electron beam absorbed current, would contribute the remaining ∼2% of *A*, we have *A*_th_ ≈ 1 – *R* when the transmittance of electrons is zero (*T* = 0) in a system. That is, *A*_th_ is nearly complementary to *R*. Thus, 1 – *A*_th_ can be regarded as an ideal backscattering electron (BSE) detector with ∼2% systematic error. As shown in Figure 1c, the above statement has been experimentally verified for materials with atomic numbers (*Z*) ranging from 1 to 79. We have demonstrated that this technique can determine the effective *Z* (*Z*_eff_) of a sample.^23^ Unlike BSE detectors, which have weak responses to light elements or compounds, our ZEM technique is particularly sensitive to low *Z* materials due to its signal being complementary to that of an ideal BSE detector. ZEM also eliminates many uncertainties associated with BSE detectors and can serve as a standardization tool.^24^ This work will explore ZEM’s potential in characterizing hydrogen storage materials.

## Results/Discussion

### Quantifying Hydrogen Storage at the Nanoscale

As shown in Figure 1a,b, the ZEM measurement platform is like a bolometer. It consists of a suspended SiN_*x*_ membrane with patterned Pt wires on it. The linear resistance vs temperature of the Pt wire can serve as a thermometer to measure the temperature variation when an electron beam raster scans the sample deposited at the bolometer. An electron beam acceleration voltage (*V*_acc_) to satisfy the *T* = 0 condition can be estimated by employing Monte Carlo simulation using the CASINO program.^25 −27^ Using a stoichiometric Si_3_N_4_ nanowire as calibration, the uncertainty of *A*_th_ is estimated to be ±1%, and the accuracy of *Z*_eff_ is found to be better than 1% (see Supporting Information S1).

**Figure 2:**
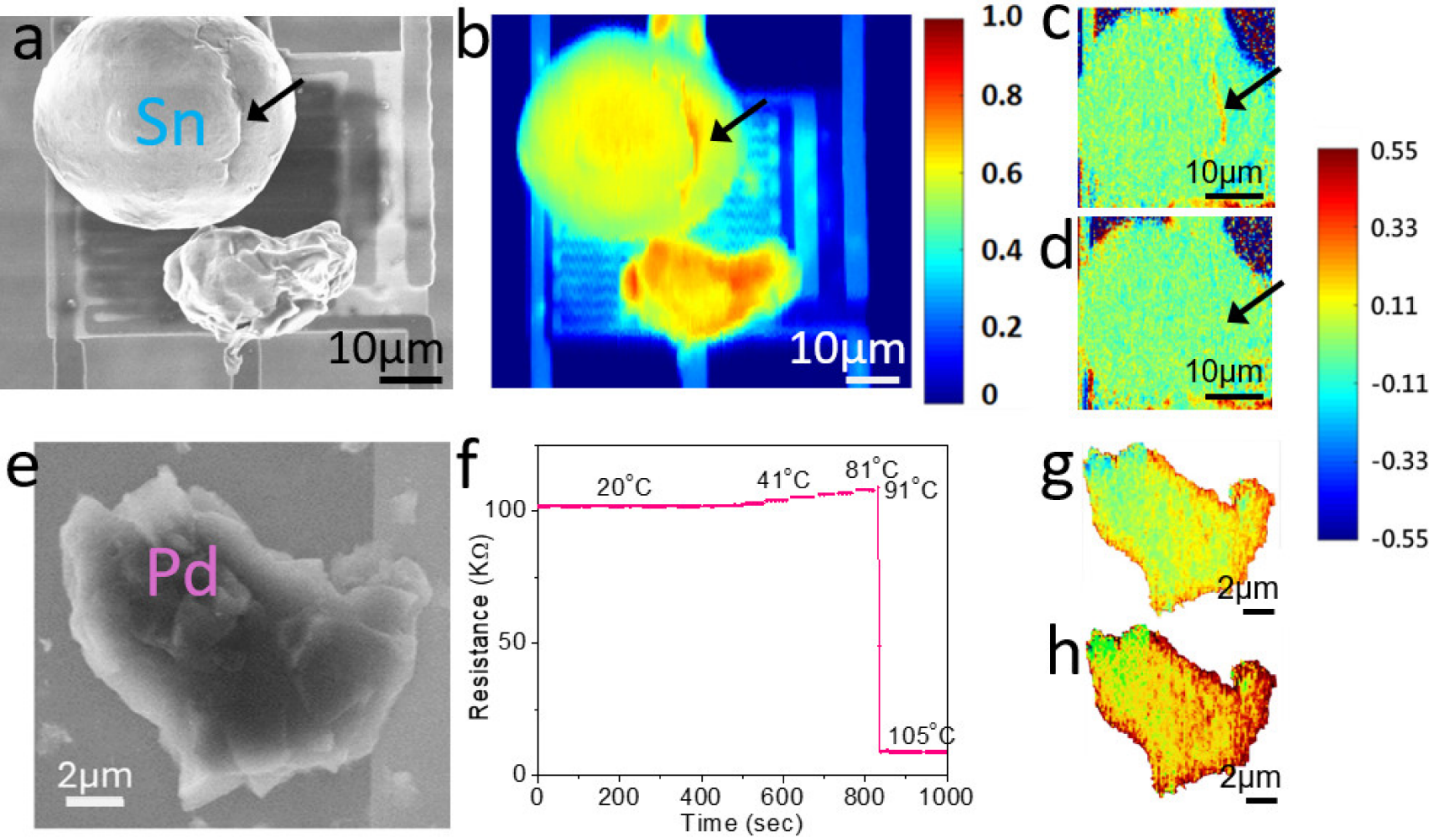
Quantifying hydrogen storage using ZEM. (a and b) An SEM image and the corresponding ZEM image of a Sn microball. Note that there is a crack (indicated by an arrow) in the microball. (c) Mapping of (*A*_th_(SnH_*x*_) – *A*_th_(Sn))/*A*_th_(Sn) after charging hydrogen and (d) after putting the sample in the SEM chamber 30 min later. Notice the disappearance of hydrogen absorption in the crack (indicated by an arrow). (e) An SEM image of a Pd micrograin. (f) Time-dependent electrical resistance measurement of a PdH_*x*_ micrograin. The temperature of the PdH_*x*_ micrograin is gradually raised until hydrogen desorption occurs at 91 °C. (g) Mapping of (*A*_th_(PdH_*x*_) – *A*_th_(Pd))/*A*_th_(Pd) after charging hydrogen. Here, the average hydrogen content *x* = 10%. The color bar is for (c, d, g). (h) Enhanced contrast of (g) to show the correlation between *x* and the grain boundaries shown in (e).

We first chose Sn as a controlled sample to test ZEM’s capability to detect hydrogen. Figure 2a, b shows a Sn microball’s SEM and corresponding *A*_th_ image. Unlike the SEM image, where brightness and contrast can be arbitrarily adjusted by the user, the brightness and contrast of *A*_th_ images are fixed, allowing an absolute color bar of absorbance for each ZEM image. This advantage of ZEM thus enables quantitative *Z* analysis.

Because the *Z* of Sn is *Z*_Sn_ = 50, the *Z*_eff_ of SnH_*x*_ would be *Z*_SnH_ = (50 + *x*)/(1 + *x*) < *Z*_Sn_. From Figure 1c, the hydrogen absorption would reduce *Z*_eff_, and correspondingly, displays an enhanced *A*_th_. On the other hand, because it has been known that surface morphology, such as tilted facets, edges, or voids, would also alter the signals of ZEM,^23^ we employ a normalized quantity (*A*_th_(SnH_*x*_) – *A*_th_(Sn))/*A*_th_(Sn) to eliminate their effects and to highlight the *Z*_eff_ variations (see Supporting Information S2). Figure 2c displays the variations of (*A*_th_(SnH_*x*_) – *A*_th_(Sn))/*A*_th_(Sn) of the Sn microball. Note that only the crack region displays hydrogen absorption. Interestingly, Figure 2d shows that the amount of hydrogen in the crack decreases after 30 min, indicating desorption of hydrogen occurs in the SEM chamber (vacuum pressure <10^–5^ mbar). Because Sn is not a hydrogen storage material and cracks are known to physically absorb hydrogen stronger than other regions, the result confirms ZEM’s capability in detecting hydrogen in materials.

Next, we choose palladium (Pd) as a representative hydrogen storage sample. Pd has been a prototypical metal-hydride system in virtually every aspect of the envisioned hydrogen economy, including hydrogen purification, storage, detection, and fuel cells.^28^ Pd exhibits fast dissociation kinetics of molecular hydrogen at its surfaces, showing high bulk diffusivity of atomic hydrogen at room temperature and atmospheric pressure. Thus, Pd has been used as a catalyst to facilitate the uptake and dissociation of hydrogen in other metal hydrides, reducing their hydrogenation time.^28^ The bulk PdH_*x*_ phase diagram exhibits a miscibility gap below 290 °C between a diluted α phase with *x* < 0.017 and a concentrated β phase with *x* > 0.6 at room temperature. On the other hand, because of the accompanied lattice distortion during hydrogenation processes, the elastic energy barrier of Pd is size- and shape-dependent, leading to distinct thermodynamic phenomena at the nanoscale.^29 −32^ Unfortunately, our understanding of hydrogen absorption and desorption in Pd remains incomplete, primarily because vacancies and other defects are nearly undetectable using conventional nanoscale characterization techniques. Moreover, the relationship between defect formation and repeated hydrogen charging/discharging processes is unknown. These challenges make Pd an ideal candidate for ZEM characterization, offering a promising approach to gaining deeper insights into nanoscale hydrogenation dynamics.

Similar to the Sn microball, we repeated the experiment on a Pd micrograin, whose SEM image is shown in Figure 2e. Because Pd is a metal, whereas PdH_*x*_ is a semiconductor, an independent resistance measurement of a Pd grain can be carried out simultaneously to monitor its hydrogen uptake. Figure 2f shows that the high resistance state can be kept for a long time in a vacuum chamber until the sample is heated beyond 91 °C to discharge the hydrogen. Figure 2g displays the mapping of (*A*_th_(PdH_*x*_) – *A*_th_(Pd)_0_)/*A*_th_(Pd)_0_ (where *A*_th_(Pd)_0_ is the of a pristine Pd, in which no hydrogen absorption has occurred) of a Pd micrograin. The relation of (*A*_th_(PdH_*x*_) – *A*_th_(Pd)_0_)/*A*_th_(Pd)_0_ vs *x* established by ZEM enables us to obtain *x*’s distribution (see Supporting Information S3). Although high *x* appeared at the edges of Figure 2g are probably due to artifacts of imaging processing (originating from scanning drift and the lattice expansion of PdH_*x*_ that make precisely locating the edges of the micrograin difficult,^33^ averaging the whole area of the micrograin gives *x* = 10%. Curiously, *x* is inhomogeneously distributed; some regions appear to absorb hydrogen with considerably higher *x* ∼ 36% while others exhibit *x* ∼ 0%. After enhancing the contrast shown in Figure 2h, we observe correlations between the *x* and the grain boundaries. Because of a significant volume fraction of grain interfaces, the hydrogen solubility in the nanocrystalline Pd film is much higher than in a conventional bulk Pd.^29,32,34−36^ Besides, surface effects, defects, and internal stresses at the grain boundaries may influence hydrogen solubility as well,^6,29,32,36^ resulting in the observed *x* = 36%. The determination of *x* and its correlation with grain boundaries thus demonstrate ZEM’s capability to detect hydrogen at the nanoscale.

### The Evolution of Hydrogens and Defects via Cyclic Charging–Discharging

Because *A*_th_(Pd)_0_ is not always experimentally available, we choose *A*_th_(Pd)_0_ = 0.696 obtained from CASINO simulation in the following works. Experimentally, we will discharge a Pd micrograin first before undergoing multiple hydrogen charging–discharging cycles. The *A*_th_’s from a selected flat region of the Pd micrograin is used to represent the *Z* variations. A flat region will enable its absolute *A*_th_ to be determined without unwanted perturbations from tilted facets, edges, cracks, or other surface morphological effects.^23^

**Figure 3:**
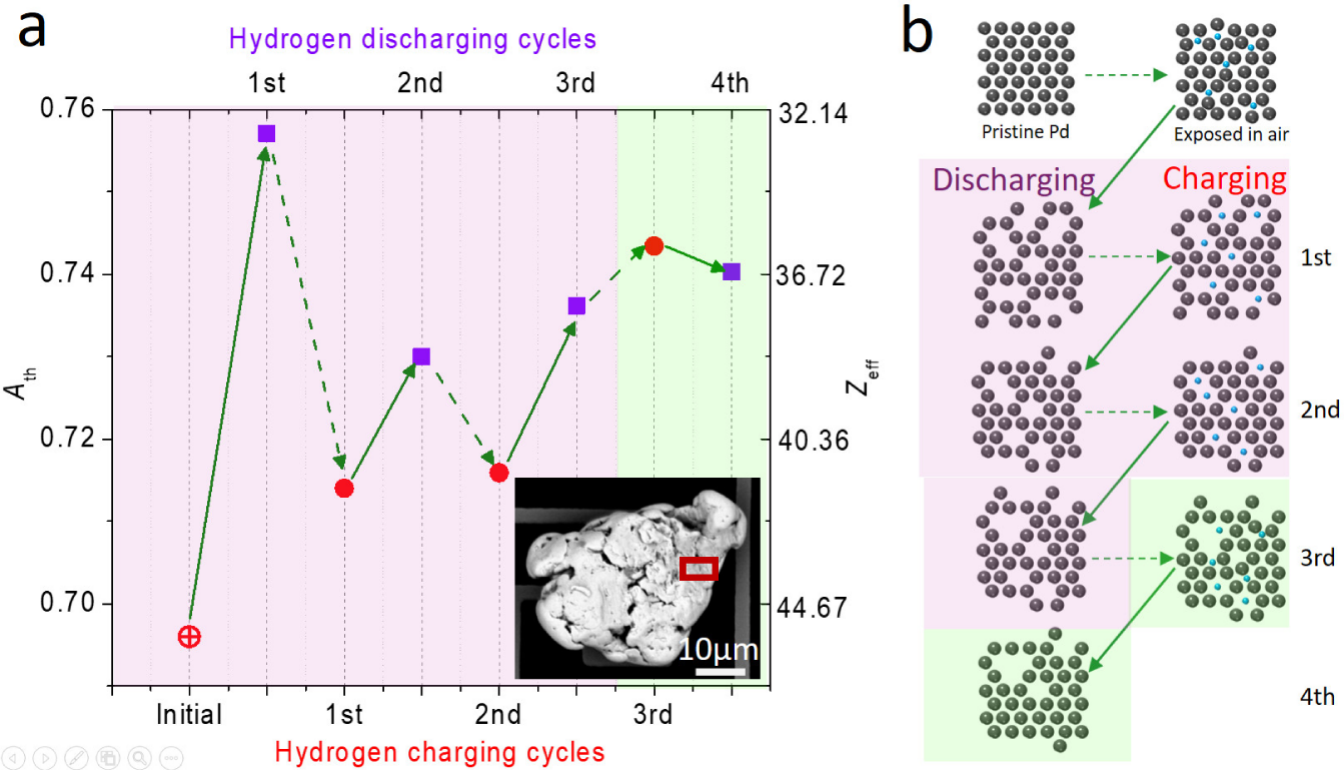
Evolution of *A*_th_ and *Z*_eff_ for multiple hydrogen charging–discharging cycles. (a) The evolution of *A*_th_ (left axis) and *Z*_eff_ (right axis) for a Pd micrograin undergoing three hydrogen charging–discharging cycles. The discharged (solid arrows) and charged (dashed arrows) processes would result in Pd (purple squares) or PdH_*x*_ (red circles). The insets show the SEM image of the Pd micrograin. The *A*_th_ of a pristine Pd, determined by CASINO simulation (*A*_th_(Pd)_0_ = 0.696), is denoted as the open-crossed circle. The measurement uncertainties of *A*_th_ is estimated to be ±1%. (b) Schematic illustrations of the hydrogen uptake and defect formation processes during the charging–discharging cycles. Here, Pd and hydrogen atoms are denoted as dark gray and blue circles, respectively. Two distinct phenomena, respectively showing *A*_th_(PdH)_m_ < *A*_th_(Pd)_m_ (*m* = 1, 2, 3) and *A*_th_(PdH)_3_ > *A*_th_(Pd)_4_ are denoted by the shaded purple and green regions, respectively. The former suggests that most hydrogens fill the defects after charging (leading to an increase of *Z*_eff_) while the latter indicates hydrogens react with Pd (leading to a decreased *Z*_eff_). Defects healing after discharging is observed in both cases.

Our initial observation in Figure 3a reveals that the *A*_th_ of the first discharged Pd (*A*_th_(Pd)_1_ = 0.757) is much higher than that of *A*_th_(Pd)_0_, which is equivalent to a reduction of *Z*_eff_ from 46 to 32.6. To explain the result, we note that superabundant vacancies,^37−39^ vacancy-ordered phases,^38,40,41^ and reduction of vacancy formation enthalpy after hydrogenation^42^ have been found in bulk Pd. Because monovacancies (*Z*_Vac_ = 0) and other forms of defects would enhance *A*_th_ and, correspondingly, reduce *Z*_eff_ of Pd (see Supporting Information S4), the reduction of *Z*_eff_ beyond its initial value *Z*_Pd_ = 46 after the first discharging shown in Figure 3a suggests the presence of defects.

From *Z*_PdH_ = (46 + *x*)/(1 + *x*) for PdH_*x*_ and *Z*_PdVac_ = 46/(1 + *v*_eff_) for Pd containing *v*_eff_ defects in a unit cell (assuming *Z*_Vac_ = 0 for a monovacancy and other forms of defects, here *v*_eff_ is the effective defect density), we find that while *x* varies at around 11% for the first two cycles in Figure 3a, *v*_eff_ reaches the highest value of 40% at the first cycle and reduces to *v*_eff_ = 25% at the third cycle. The estimated *v*_eff_ is higher than those reported in the literature because it contains contributions from other forms of defects (including voids, see Supporting Information S4). Based on the known result that hydrogens in PdH_*x*_ randomly occupy the lattice interstices,^43^ we illustrate the lattice model of the initially charged PdH_*x*_ in the first row of Figure 3b and the formation of defects in the second row of Figure 3b for the discharged Pd model.

When repeating hydrogen charging, Figure 3a shows *A*_th_(PdH)_m_ < *A*_th_(Pd)_m_ (*m* = 1, 2, 3) and oscillations of *A*_th_ are observed. Naively, if the charged hydrogens continue to fill the lattice interstices and become PdH_*x*_, the *Z*_eff_ should have decreased and an opposite effect (i.e., *A*_th_(PdH)_m_ > *A*_th_(Pd)_m_) should have been observed. To explain the data, hydrogens (*Z*_H_ = 1) must fill the defects (*Z*_Vac_ = 0) so as to increase the *Z*_eff_ and to be consistent with the observation of *A*_th_(PdH)_m_ < *A*_th_(Pd)_m_ (*m* = 1, 2, 3). The corresponding lattice models are illustrated in the second to the fourth rows of Figure 3b.

Curiously, we observe *A*_th_(Pd)_first_ > *A*_th_(Pd)_2 rd_ ∼ *A*_th_(Pd)_third_ in Figure 3a, indicating that *v*_eff_ is reduced after the second discharging. The unusual phenomenon has been suggested by theoretical calculations that voids in Pd are thermodynamically unstable due to the loss of configurational entropy upon coalescing of monovacancy complexes.^44^ In fact, stable voids in Pd are attributed to other factors, such as strain induced by hydrogen dissolution or hydrogen-enhanced local plasticity.^44,45^ Because the time scale for conducting our experiment suggests that the observed *v*_eff_ in the Pd micrograin remains relatively stable at least for days, Figure 3a indicates that *v*_eff_ would change only when the Pd undergoes cyclic charging–discharging processes. During the hydrogen charging process, lattice strain would increase, facilitating the decomposition of voids into monovacancies via defect migration.^46^ Thus, *A*_th_(Pd)_first_ > *A*_th_(Pd)_2 rd_ ∼ *A*_th_(Pd)_third_ suggests a healing process of voids, as illustrated in Figure 3b.

The healing process of voids may also explain why the ratio of vacancy to hydrogen concentration is high (*v*_eff_*/x* = 3.6) at the beginning and decreases to *v*_eff_*/x* = 0.85 after the third cycle. It has been suggested that up to six hydrogen atoms can be captured by a monovacancy,^47,48^ whereas the number of hydrogens captured by a void should be less due to the reduced surface-to-volume ratio. Because the initial *v*_eff_ contains a larger contribution of voids that tend to overestimate *v*_eff_ by a factor of 3.5 (see Supporting Information S4), the reduction of *v*_eff_/*x* also suggests the decomposition of voids into monovacancies during the cyclic hydrogen charging–discharging processes.

We notice that the oscillation amplitude [*A*_th_(Pd)_m_ – *A*_th_(PdH)_m_ (*m* = 1, 2, 3)] gradually decreases for increasing m and *v*_eff_ becomes more for *m* > 2. After the third cycle, an opposite effect, *A*_th_(PdH)_3_ > *A*_th_(Pd)_4_, is observed. The result indicates that hydrogens no longer fill the defects, but they react with Pd instead. Currently, it is not known whether the Pd micrograin gradually loses its capability to uptake hydrogen or there is a balance between the two opposite effects (i.e., *A*_th_(PdH)_m_ < *A*_th_(Pd)_m_ and *A*_th_(PdH)_m_ > *A*_th_(Pd)_m_).

Because similar phenomena, including a jump of *A*_th_ in the first discharging and oscillations of *A*_th_’s at the later cycles, are observed in Region II and in another Pd micrograin (see Supporting Information S5 and S6), they could suggest a universal defect formation and healing phenomenon.

Compared with other techniques, the differential thermal expansion method relies on X-ray or TEM diffraction to determine the lattice contraction, which is sensitive to monovancancies only. PAS depends on the lifetime measurements of positrons, and the estimation of defect concentrations relies on the parameters of the trapping model, which has been known to be inadequate for materials with superabundant vacancies.^49,50^ Thus, the excess *v*_eff_ observed by ZEM indicates that previous characterization tools could have missed voids, cracks, and cliffs.

Because the existing nanoscale hydrogen characterization tools, such as SIMS or APT, are constrained by their destructive detection methods, it is impossible for them to reveal the cyclic charging–discharging process shown in Figure 3a. Thus, the unusual behavior shown in Figure 3a has never been reported to our knowledge.

### The Depth Profile of Defect Density

**Figure 4:**
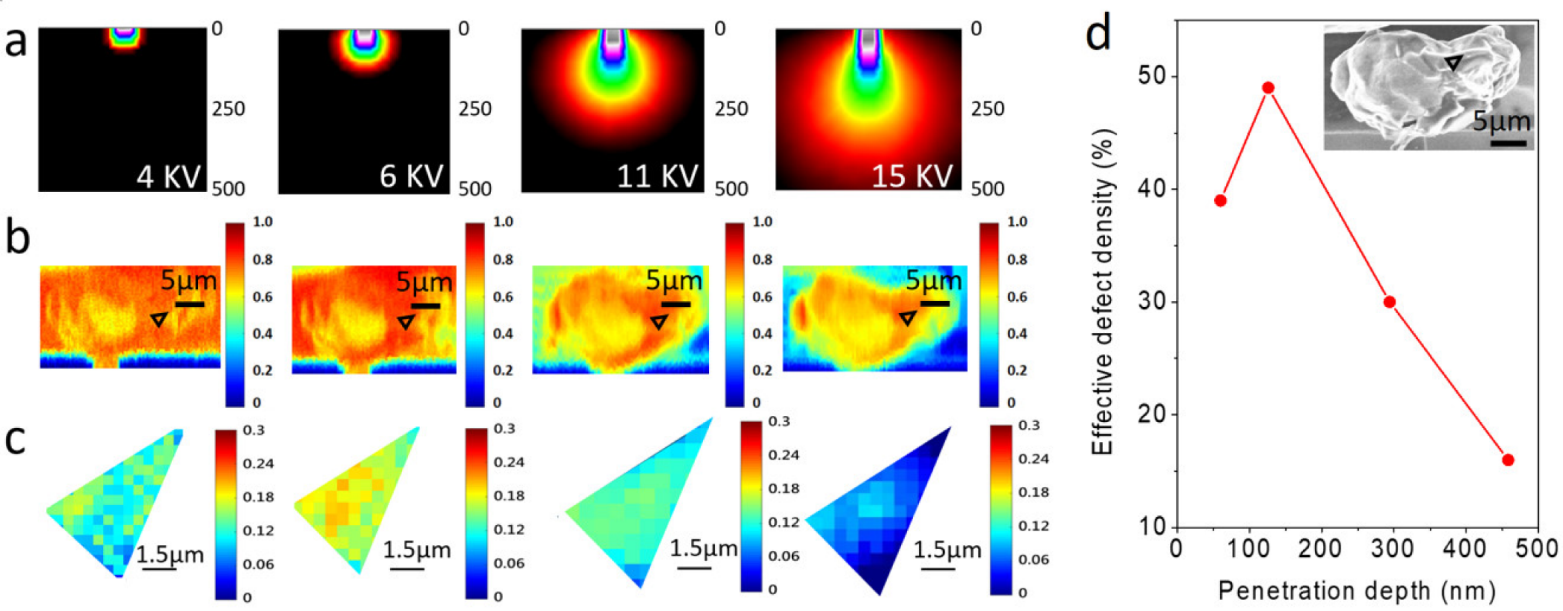
Depth profile of the effective defect density. (a) Simulated electron energy distribution under different *V*_*acc*_’s (b) ZEM images of a Pd micrograin for different *V*_*acc*_’s. (c) Mapping of (*A*_th_(PdH_*x*_) – *A*_th_(Pd))/*A*_th_(Pd) on the flat region (denoted by the black triangles in (b)) of the Pd micrograin. (d) Effective defect density vs penetration depth of the flat region. The inset shows the SEM image of the Pd micrograin. The selected flat region for analysis is denoted as the black triangle. The *v*_eff_’s depth profile can be associated with the hydrogen uptake, hydrogen diffusion, defect creation, and defect healing processes in Pd.

Because varying the acceleration voltage (*V*_acc_) of an electron beam is known to change the penetration depth (*d*) in a material,^51^ ZEM can profile the defect density at different depths (see Supporting Information S7). Figure 4a shows the simulated interacting profiles of an electron beam accelerated at different *V*_acc_’s and the associated *d* in Pd. We can see that increasing *V*_acc_ from 4 kV to 15 kV would increase the penetration depth from 60 to 458 nm, allowing us to probe deeper *A*_th_’s beneath the surface. Figures 4b,c show the ZEM images at different *V*_acc_’s and the corresponding flat region selected for *Z* analysis. Interestingly, we find *v*_eff_ increases from 39% at *d* = 60 nm to 49% at *d* = 126 nm and decreases to 16% at *d* = 458 nm, as shown in Figure 4d. As mentioned above, the *v*_eff_’s observed here may also contain contributions of monovancancies, voids, cracks, and other defects. Because hydrogen is known to reduce the defect formation energy and facilitate the motion of dislocations,^52−54^ the observed vacancy depth profile qualitatively agrees with the picture that hydrogen diffuses from the surface to the interior of Pd, creates various forms of defects, and the healing of the voids may further alter the distribution of *v*_eff_ in the depth profile.

### The Causality between Hydrogen Storage and Defect Formation

The relation between hydrogen storage and defect density has been established in bulk Pd, Nb, Ni, Co, and Fe,^55^ in which the defect density is measured after a hydrogen charging–discharging cycle is applied. However, the lack of time-sequential analysis and the poor spatial resolution in previous works have prohibited the establishment of causality between hydrogen storage and defect formation. Here, we are particularly curious about the question: After the first cycle, do hydrogens continue to create defects in Pd, or do the preexisting defects become the primary sites attracting hydrogen in subsequent cycles?

**Figure 5:**
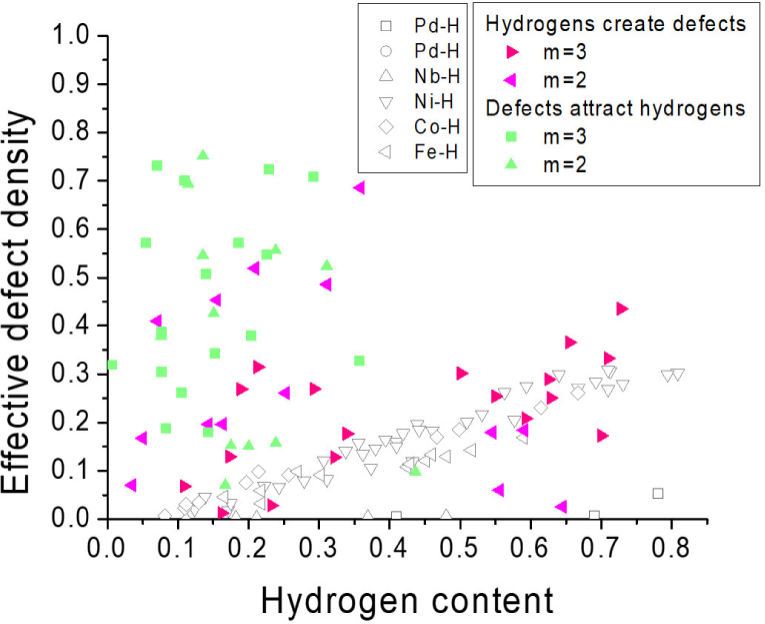
Causality between hydrogen storage content and the effective defect density. To investigate the causality, the data for “hydrogen creates defects” are obtained from the m^th^ charging and discharging cycle in Figure 3, and the correlation coefficient = 0.4–0.64 is found. On the other hand, the data for “defects attract hydrogens” are obtained from the m^th^ discharging cycle and the (m + 1)^th^ charging cycle in Figure 3, and the correlation coefficient = 0.09–0.18 is obtained. Data established from bulk materials (open symbols) are also shown for comparison.^55^ Our result suggests that hydrogenation creates defects, but defects do not necessarily attract hydrogens in Pd.

To answer the former question, we have applied pixel-by-pixel correlation analysis for ZEM images of hydrogen content and defect density at the m^th^ cycle (*m* = 2, 3), in which hydrogen charging occurs first and discharging happens later. Interestingly, Figure 5 shows that the correlation is strong (correlation coefficient = 0.4–0.64) and agrees with previous results observed in bulk metal hydrides.

To answer the latter question, we investigate the defect density at the m^th^ cycle and the hydrogen content at the (m + 1)^th^ cycle, in which the discharging occurs earlier than charging. Figure 5 shows a weaker correlation (correlation coefficient = 0.09–0.18), indicating that a significant number of defects exist but cannot attract the same amount of hydrogens. Note that the result does not contradict the model shown in Figure 3b, in which we find that some, but not all, defects are filled with hydrogens.

The distinct correlations establish the causality that hydrogenation creates defects, but defects do not necessarily attract hydrogens in Pd. At first glance, this might imply that *v*_eff_ would continually increase with successive hydrogen charging–discharging cycles; however, this is certainly not the case. Thus, one possibility is the defect healing effect that decreases *v*_eff_. Another possibility is the correlation of hydrogen-creating-defects may become weaker for the (*m* > 4)^th^ cycles. Both of them have been observed in Figure 3.

## Conclusion

Combining the new results provided by the ZEM, we now have a more complete understanding of hydrogen uptake and defect formation processes in Pd. Hydrogens enter Pd’s surface and create various forms of defects. While the average hydrogen uptake in a PdH_*x*_ micrograin is *x* ∼ 10%, its distribution is highly inhomogeneous, reaching more than *x* = 36% at grain boundaries or defects. Hydrogens’ diffusion into Pd’s interior also generates a large defect density gradient, reaching *v*_eff_ = 49% at 126 nm beneath the surface and *v*_eff_ = 16% in the interior of Pd. While some of the defects may attract hydrogens during charging, voids may decompose into monovacancies due to their thermodynamic instability. Defects are considered the precursor of hydrogen embrittlement;^3,4,52 −54,56,57^ however, very few existing tools can characterize them at the nanoscale. These results demonstrate ZEM’s potential for material characterizations, especially for unraveling the role of hydrogens and defects in the science of catalysis and sustainable energy.

## Methods

### Materials

Palladium (Pd) fine powders with 99.995% purity were obtained from Sigma-Aldrich. The powders were dispersed in alcohol and deposited onto a ZEM measurement platform. Hydrogen discharging was performed within the SEM chamber by applying a high Joule heating current to the ZEM platform, raising its temperature above 120 °C for 30 min. For hydrogen charging, the Pd sample was exposed to 99.999% pure hydrogen at a pressure of 1.5 bar for 1 h in a custom-built chamber. Following this process, the sample was rapidly transferred to the SEM chamber for analysis.

### Measurements

ZEM measurements were performed using a Zeiss Auriga SEM. A LabView program controlled the raster scanning of a focused electron beam across the sample. A Keithley 224 current source supplied DC currents, while voltage responses from the ZEM platform were recorded using a Wheatstone bridge setup with an HP/Agilent 34970A data acquisition system. The ZEM images of discharged Pd micrograin were scanned twice to obtain the normalized variation of *A*_th_, which minimizes the unwanted effects of structural perturbations.

### Simulations

The simulations were performed using the CASINO Monte Carlo program (Version 3.3.0.4) with the MONSEL Defaults model. This model integrates the Browning approach for calculating the Mott elastic scattering cross-section and employs the Joy and Luo model, further modified by Lowney, to compute inelastic scattering energy loss (*dE*/*dS*). In each simulation, 100,000 electrons were input with varying electron beam diameters and acceleration voltages (*V*_acc_ = 1–20 kV). This setup allowed for the determination of interaction volume, reflectance (*R*), transmittance (*T*), absorbance (*A*), the effective defect density (*v*_eff_), and the penetration depth (*d*) based on the specified parameters. Please see Supporting Information for more details.

## Supporting Information Available

Experimental procedures and the accuracy and precision of *A*_th_ measurements; image processing; the relation between *A*_th_, *Z*_eff_, and hydrogen content (*x*); Monte Carlo simulation of the absorbance of voids; the evolution of hydrogens and defects in Region II; the evolution of hydrogens and defects of another Pd micrograin; and the electron beam’s penetration depth (*d*) vs *V*_acc_ (PDF)
