# Supplementary material for: Quantitatively Profiling the Evolution of Hydrogen Storage and Defect Healing Processes in Palladium at the Nanoscale
Source: ACS Nano. 2025 Mar 4;19(10):10070–7. doi: 10.1021/acsnano.4c16841 (PMC11924336; doi:10.1021/acsnano.4c16841)
Supplement: Supplementary file 1 — nn4c16841_si_001.pdf [file nn4c16841_si_001.pdf]

Supporting Information

# Quantitatively profiling the evolution of hydrogen storage and defect healing processes of palladium at the nanoscale

Yu-Cheng Chiu,[1,2] Bo-Yi Chen,[1] Chin-Chia Hsu,[1,2] Chia-Wei Tsai,[1,2] Shih-Ming Wang,[1,2] I-Ling Chang,[2] and Chih-Wei Chang[1,3*]

[1] Center for Condensed Matter Sciences, National Taiwan University, Taipei 10617, Taiwan

[2] Department of Mechanical Engineering, National Cheng Kung University, Tainan 70101, Taiwan

[3] Center of Atomic Initiative for New Materials (AI-MAT), National Taiwan University, Taipei, 10617, Taiwan

## S1. Experimental procedures and the accuracy and precision of $A_{th}$ measurements.

Palladium (Pd) fine powders with 99.995% purity were purchased from Sigma-Aldrich. They were dispersed in alcohol and then dropped on a ZEM measurement platform. The hydrogen discharging process was made in the SEM chamber by applying a large Joule heating current to our ZEM platform to increase its temperature beyond 120°C for 0.5 hours. The hydrogen charging of Pd was carried out at 1.5 bar, under 99.999% pure hydrogen pressure, for 1 hour in our homemade chamber. Then, the sample was quickly transferred to our SEM chamber.

Similar to our previous work[1], the ZEM measurements were conducted using Zeiss Auriga SEM. A LabView program was used for raster-scanning a focused electron beam on a sample, while a Keithley 224 current source was used for supplying DC currents, and an HP/Agilent 34970A was used to read the voltage responses from the ZEM platform using a Wheatstone bridge.

Given that our ZEM signals are always normalized to $A_{th}$ of a 400 nm thick $SiN_x$ film, the $SiN_x$ film effectively serves as the reference standard for calibration. However, because of the non-stoichiometric $SiN_x$, further calibration is necessary to precisely determine its $Z$. Figure S1(a & b) shows a single-beam bolometer with a stoichiometric $Si_3N_4$ ($Z$=10) nanowire deposited at its center. The single-beam bolometer allows all relevant physical quantities to be precisely measured. Figure S1(c) shows $A_{th}$'s of regions randomly selected from a $SiN_x$ film. The measurement uncertainty is less than 1% for each image. Because of the presence of edges or tilted facets that would reduce $A_{th}$, the strongest $A_{th}$'s are selected from Fig. S1(c) to represent the intrinsic $A_{th}$ of the $Si_3N_4$ nanowire. The results are compared with those of $SiN_x$ film, and we find that $A_{th}$($Si_3N_4$ nanowire)/$A_{th}$($SiN_x$ film)=1.0084. From the CASINO simulation, we have

$A$=0.89 for $Si_3N_4$ ($Z$=10). Thus, it suggests that the low-stress $SiN_x$ film is in fact Si-rich and exhibits $Z$=11.3 (x=0.63). In addition, because $A_{th}/A$=98% of the $SiN_x$ film has been established in our previous work and by P. Y. Yuan *et al.*[1,2], the accuracy of ($A_{th}$-$A$)/$A$ is estimated to be 2%. This calibration procedure enables more accurate determinations of $Z$ for other materials.

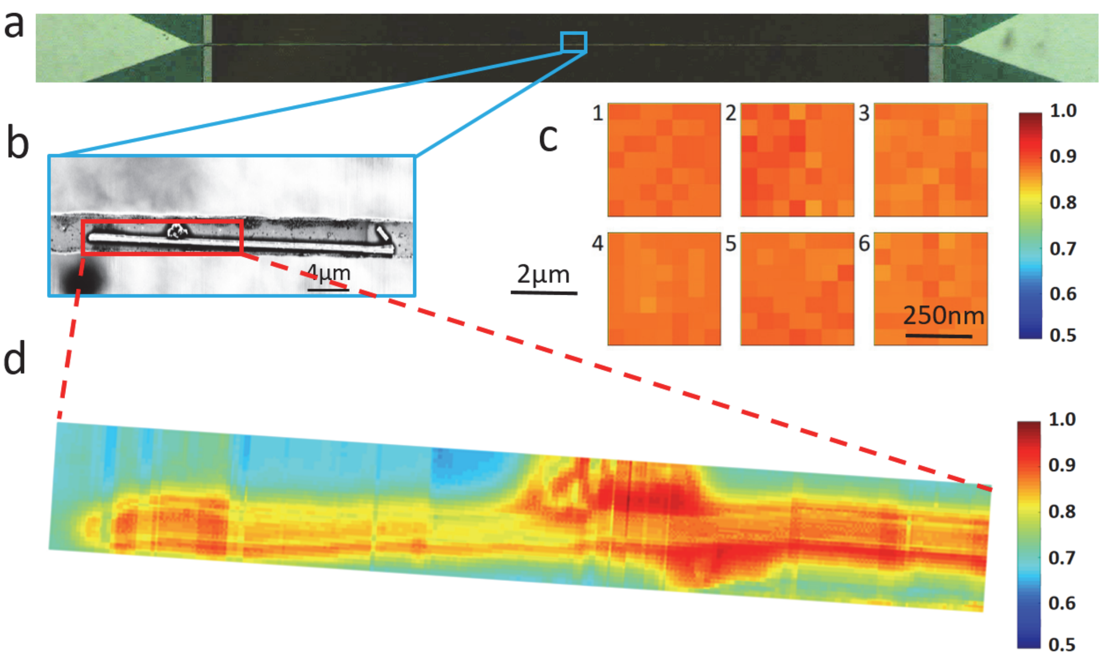


**Fig. S1. Determining the accuracy and precision of $A_{th}$ measurements.** (a) An optical image of a 1450μm-long $SiN_x$ beam with 50nm Pt film deposited on it. The setup is used for calibration of the $A_{th}$ of a $Si_3N_4$ nanowire. (b) An enlarged SEM image of the center of a $Si_3N_4$ nanowire. (c) $A_{th}$'s of regions are randomly selected from a flat $SiN_x$ film. For each 7×7 area, the measurement uncertainty is less than 1%. (d) $A_{th}$ mapping of the $Si_3N_4$ nanowire. Its signals are compared with those of $SiN_x$ film. We find $A_{th}$($Si_3N_4$ nanowire)/$A_{th}$($SiN_x$ film)=1.0084.

We have also investigated the robustness of $A_{th}$ measurements for prolonged scanning. As shown in Fig. S2(a), repeated and prolonged SEM scanning under low $V_{acc}$ is known to cause hydrocarbon deposition on a sample. As displayed in Fig. S2(b), we have found that the effect is not pronounced for the $A_{th}$ measurement of the Ag nanowire, Pt film, and $SiN_x$ membrane shown in Fig. S2(a) when operating $V_{acc}$ = 5KV for 4 hours (which is about the accumulated scanning time we conducted for the Pd micrograin undergoing multiple hydrogen charging-discharging cycles). From Fig. S2(b), the largest variation of $A_{th}$ is found in the Ag nanowire, whose monotonic 5% increase can be attributed to the hydrocarbon deposition. On the other hand, $A_{th}$'s of the Pt film and the $SiN_x$ membrane show ~1.7% variations that are much smaller than what we observe in Fig. 3 of the main text.

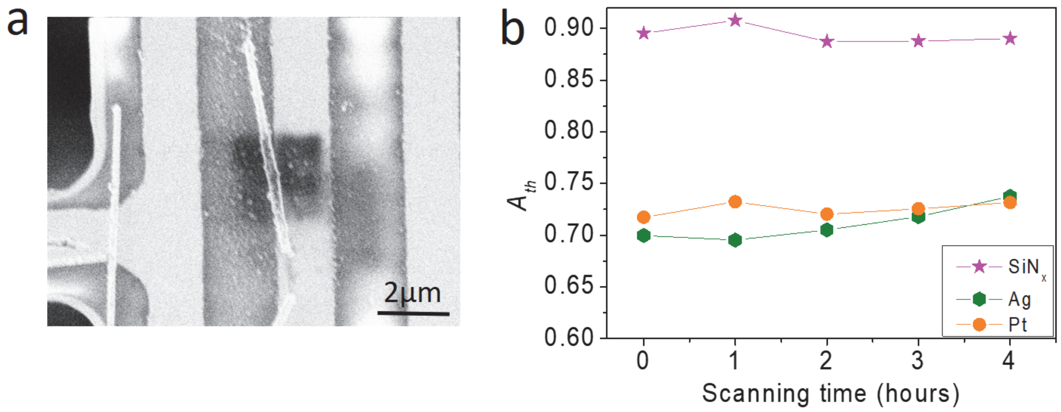


**Fig. S2. Effects of prolonged $A_{th}$ measurements.** (a) SEM image of an area (the center dark square) after prolonged $A_{th}$ measurements. (b) The corresponding $A_{th}$ variations of the Ag nanowire, the Pt film, and the $SiN_x$ membrane shown in (a).

## S2. Image processing

The processes of obtaining the normalized variation of $A_{th}$ images are shown in Fig. S3. Here the ZEM images of discharged Pd micrograin are scanned twice [respectively labelled as Pd(1) and Pd(2)], and the image of [Pd(2)−Pd(1)]/Pd(1) is obtained to highlight the relative $A_{th}$ variations.

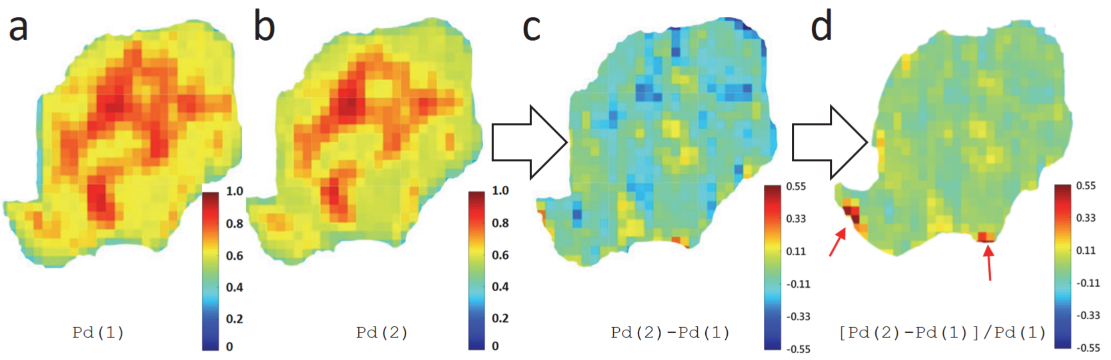


**Fig. S3. Image processing procedures.** (a & b) ZEM images of a Pd micrograin being scanned twice, whose $A_{th}$ images are labelled as Pd(1) and Pd(2), respectively. Here the crack regions are displayed as red structures. (c) The image of Pd(2)−Pd(1). (d) The image of [Pd(2)−Pd(1)]/Pd(1). Note that the crack regions are almost invisible here. Due to the uncertainties in locating the edges, the image artifacts that appear at the edges are denoted by the red arrows in (d).

Note that the crack regions [displayed as red structures in Figs. S3(a & b)] almost disappear in Fig. S3(d), which justifies that our method can minimize the structure perturbations. However, because of the scanning drift and other factors (such as lattice expansion in $PdH_x$), it is not easy to precisely locate the edges of a sample, resulting in

some image artifacts appear at the edges [denoted as red arrows in Fig. S3(d)].

## S3. The relation between $A_{th}$, $Z_{eff}$, and hydrogen content ($x$)

The principle of ZEM is based on the empirical result shown in Fig. 1(c) of the main text. Because of $A_{th}/A$=98%, the measured $A_{th}$ agrees with that of CASINO simulation very well. The result of Fig. 1(c) thus establishes a relation between $A_{th}$ and $Z_{eff}$. For $PdH_x$, we can employ CASINO simulation to obtain the relation between $A_{th}$, $Z_{PdH}=(46+x)/(1+x)$, and $x$, as shown in Fig. S4(a). Similarly, the relation between $(A_{th}(PdH_x)-A_{th}(Pd)_0)/A_{th}(Pd)_0$ and $x$ can be established as well, as shown in Fig. S4(b). Note that $A_{th}(Pd)_0$=0.696 denotes $A_{th}$ of a pristine Pd, in which no hydrogen absorption has occurred.

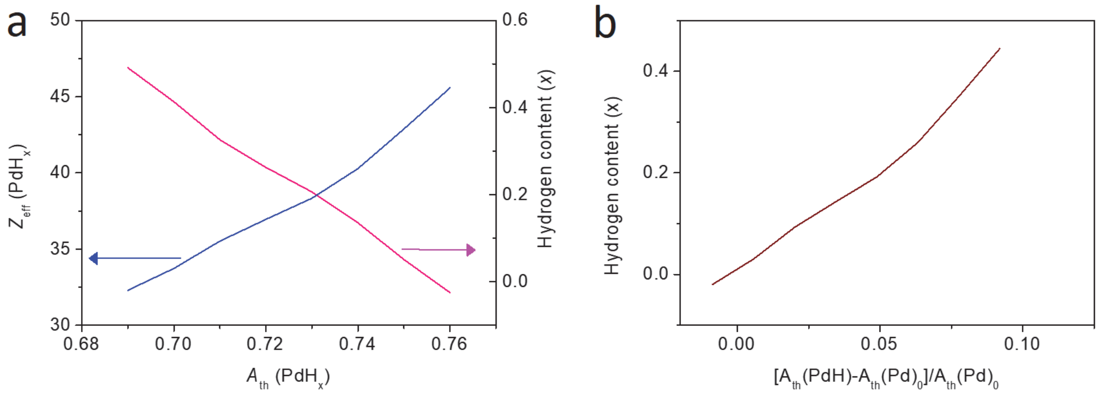


**Fig. S4. The relation between $A_{th}$, $Z_{eff}$, and hydrogen content ($x$)** (a) The relation between $A_{th}$, $Z_{PdH}=(46+x)/(1+x)$, and $x$. (b) The relation between $(A_{th}(PdH_x)-A_{th}(Pd)_0)/A_{th}(Pd)_0$ and $x$.

Although both $A_{th}$ vs $Z_{eff}$ and $A_{th}$ vs $x$ relations are approximately linear, the relation of Fig. S4 is used when estimating $x$ in our data and no linear approximation is applied.

## S4. Monte Carlo simulation of the absorbance of voids

The simulations are conducted using the CASINO Monte Carlo program (Version 3.3.0.4) using the MONSEL Defaults model[3]. The model incorporates the Browning approach for calculating the Mott elastic scattering cross-section and the Joy and Luo model[4,5], which has been further modified by Lowney[6] to calculate inelastic scattering energy loss ($dE/dS$). We typically input 100,000 electrons with various electron beam diameters and acceleration voltages ($V_{acc}$ =1~20 KV). This enables us to obtain interaction volume, reflectance ($R$), transmittance ($T$), and total absorbance ($A$) based on the input parameters.

The defect density ($\nu_{eff}$) determined by our ZEM method relies on the assumption that all vacancies are atomic scales, and its definition is to count how many atoms are

missing, on average, from a unit cell. In reality, voids or cracks may exist in the samples, and the definition of the corresponding defect density would be dissimilar. To investigate their effects, we have employed CASINO simulations on arrays of voids in $Si_3N_4$ under electron beam irradiation, as shown in Fig. S5(a). We first obtain the $A$ and follow the same guideline of our ZEM method to determine $v_{eff}$. Then we estimate how much volume of voids ($V_{void}$) is located within 90% of the interacting volume ($V_{ebeam}$) of the electron beam. The volumetric void concentration ($D_{void}$) is obtained by $D_{void} = V_{void}/V_{ebeam}$.

It must be pointed out that $D_{void}$ may not be a well-defined quantity when employing an electron beam as a probe. Furthermore, the CASINO simulation would not be accurate when the void size becomes too small. Nevertheless, it is still worthwhile to know the difference between the two concentrations. Figure S5(b) shows their ratio ($v_{eff}/D_{void}$) for different void sizes. We find that $v_{eff}$ tends to overestimate the void concentration by 150% when the void sizes are about 35nm, and the overestimation increases to 350% when the void size is reduced to 20nm.

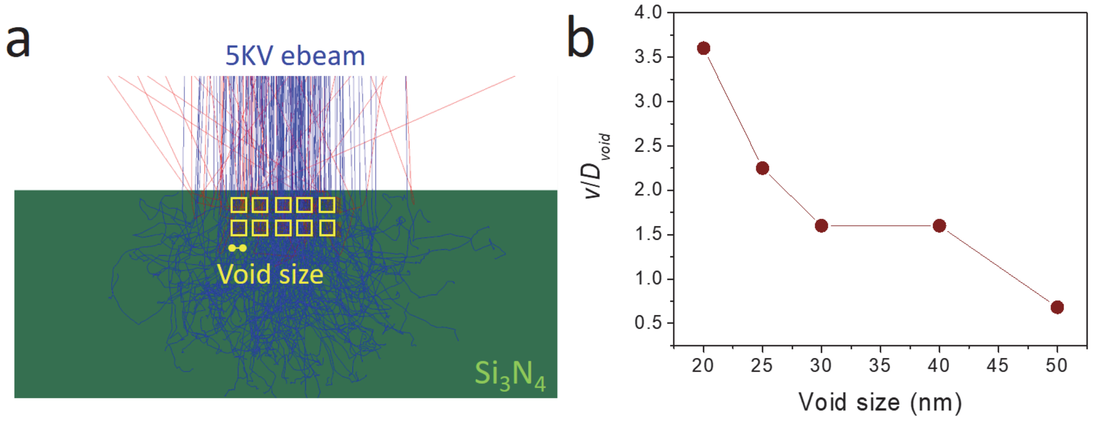


**Fig. S5. CASINO simulation of an electron beam interacting with an array of voids.** (a) Schematic illustration of a 5 KV electron beam interacting with a $SiN_x$ sample with two layers of voids. (b) $v_{eff}/D_{void}$ with different void sizes.

However, we emphasize that probing nanoscale voids and estimating their $D_{void}$ are always difficult for conventional tools, including Archimedes' method, thermal differential method, Raman spectroscopy, PL spectroscopy, etc. Although $D_{void}$ may be ill-defined at the nanoscale, the simulation results show that ZEM remains a valuable tool for estimating their abundance.

## S5. The evolution of hydrogens and defects in Region II

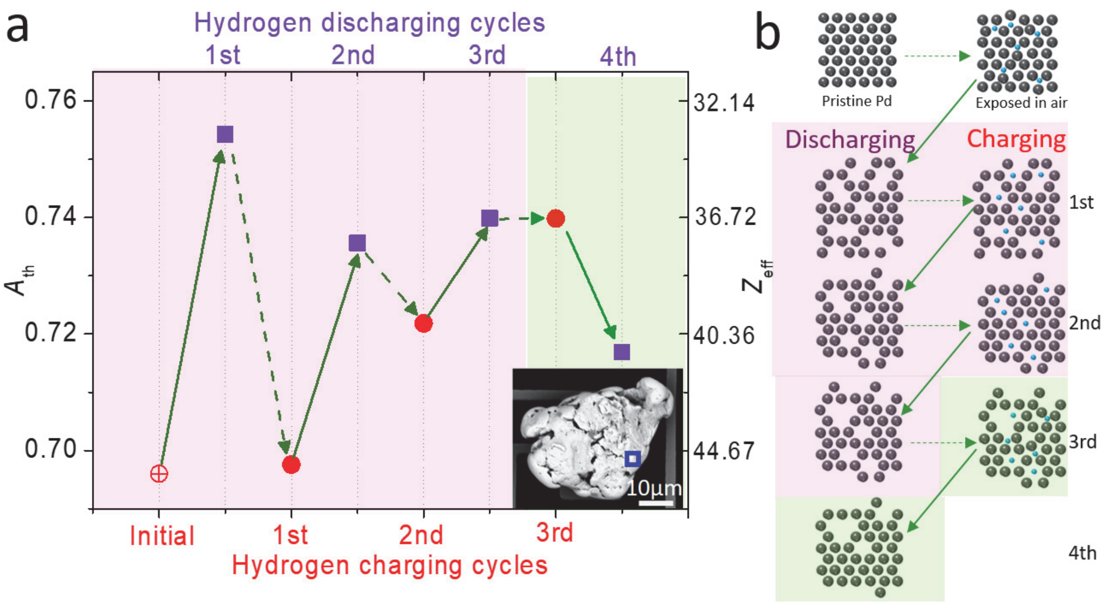


**Fig. S6. The evolution of $A_{th}$ in Region II.** (a) The Region II is denoted in the blue area shown in the inset. The discharged and charged processes are respectively denoted as solid and dashed arrows, resulting in Pd (purple squares) or $PdH_x$ (red circles). Because the $A_{th}$ of a pristine Pd is not available here, the result from the CASINO simulation ($A_{th}$=0.696, denoted as an open-crossed circle) is used as a reference. The measurement uncertainties of $A_{th}$ is estimated to be ±1%. (b) Schematic illustrations of the hydrogen uptake and defect formation processes during the charging-discharging cycles. Here, Pd and hydrogen atoms are denoted as dark gray and blue circles, respectively. The processes showing $A_{th}$(Pd)>$A_{th}$(PdH) and $A_{th}$(Pd)<$A_{th}$(PdH) are denoted by the shaded purple and green regions, respectively.

## S6. The evolution of hydrogens and defects of another Pd micrograin

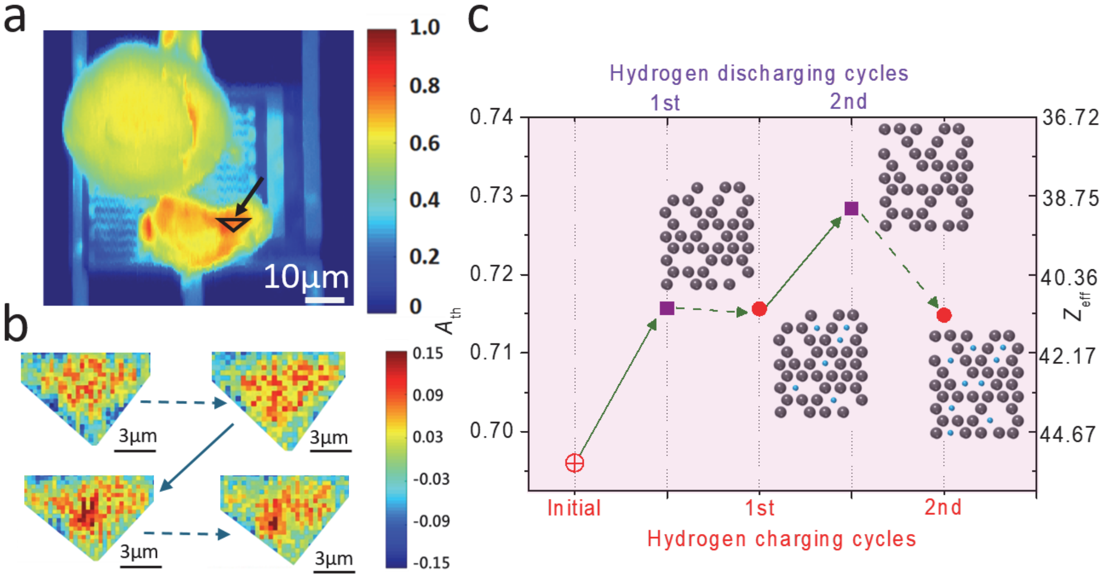


**Fig. S7. The evolution of $A_{th}$ of another Pd grain.** (a) A ZEM image (identical to Fig. 2(c)) where a flat region of the Pd micrograin (denoted by the black arrow) is used for $A_{th}$ analysis. (b) The evolution of ($A_{th}(PdH_x)-A_{th}(Pd))/A_{th}(Pd)$ of the selected region during two cycles of hydrogen discharging and

charging, where $A_{th}$(Pd)=0.696 is determined by CASINO simulation. (c) The evolution of the averaged $A_{th}$ of the selected area during the charging-discharging cycles. The measurement uncertainties of $A_{th}$ is estimated to be ±1%. The schematic illustrations of the hydrogen uptake and defect formation processes are also shown in the insets. Unlike Fig. 3 of the main text, only $A_{th}$(Pd)>$A_{th}$(PdH) processes (denoted by the shaded purple) are found here.

### S7. The electron beam's penetration depth (*d*) vs $V_{acc}$

The penetration depth (*d*) of an electron beam for a given $V_{acc}$ has been analytically studied by Kanaya and Okayama[7], in which *d* (has unit in m) can be expressed by

$$d = \frac{0.0276 m_A N_A E^{5/3}}{Z^{8/9}\rho} \qquad (1)$$

where $E$=e$V_{acc}$ is the energy of electron in KeV, $m_A$ is the atomic mass in kg, $N_A$ is Avogadro's number, $\rho$ is the density in kg/m$^3$. The *d* can also be obtained from Monte Carlo simulation by, for example, defining 10% of the incident electrons penetrate through a plane of *d* beneath the surface. Figure S8 shows the results of Eq. (1) and the CASINO simulation for Pt ($Z_{Pt}$=78). We can see that both methods are consistent with each other and their difference can be attributed to the different definitions of *d*.

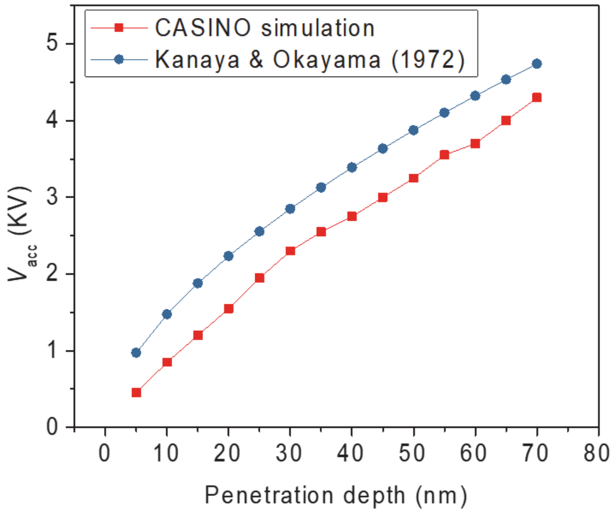


**Fig. S8. The penetration depth (*d*) vs $V_{acc}$ for Pt.** Here the analytical results from Kanaya & Okayama[7] (dark blue circles) and CASINO simulation (red square) are shown for comparison. Their difference can be attributed to different definition of *d*.

The validity of Eq. (1) and the CASINO simulation have been experimentally verified by Yuan *et al*[2].

## References

1. Lin, C. C.; Wang, S. M.; Chen, B. Y.; Chi, C. H.; Chang, I. L.; Chang, C. W. *Nano Lett.*

**2022,** 22, (7), 2667-2673.
2. Yuan, P. Y.; Wu, J. Y.; Ogletree, D. F.; Urban, J. J.; Dames, C.; Ma, Y. B. *Nano Lett.* **2020,** 20, (5), 3019-3029.
3. Demers, H.; Poirier-Demers, N.; Couture, A. R.; Joly, D.; Guilmain, M.; de Jonge, N.; Drouin, D. *Scanning* **2011,** 33, (3), 135-146.
4. Joy, D. C.; Luo, S. *Scanning* **1989,** 11, (4), 176-180.
5. Browning, R.; Li, T. Z.; Chui, B.; Ye, J.; Pease, R. F. W.; Czyzewski, Z.; Joy, D. C. *J. Appl. Phys.* **1994,** 76, (4), 2016-2022.
6. Lowney, J. R. *Scanning* **1996,** 18, (4), 301-306.
7. Kanaya, K.; Okayama, S. *J. Phys. D: Appl. Phys.* **1972,** 5, (1), 43.
